# The Role of Angiotensin-II Infusion in an Infant With Autosomal Recessive Polycystic Kidney Disease Postbilateral Nephrectomies and Refractory Hypotension in the Neonatal Period

**DOI:** 10.1155/crin/1431773

**Published:** 2025-03-24

**Authors:** Ana Nevarez Gilbert, Adrianne Rahde Bischoff, Kyle Merrill

**Affiliations:** ^1^Division of Neonatology, University of Iowa Stead Family Children's Hospital, Iowa City, Iowa, USA; ^2^Division of Nephrology, Dialysis and Transplantation, University of Iowa Stead Family Children's Hospital, Iowa City, Iowa, USA

## Abstract

Autosomal recessive polycystic kidney disease (ARPKD) is a form of hereditary cystic disease with a highly variable phenotypic expression that ultimately leads to chronic kidney disease. Severe cases may warrant surgical intervention with unilateral or bilateral nephrectomy to alleviate thoracic and abdominal compression from massive nephromegaly. Hypotension has been identified as a potential complication following nephrectomy in pediatric patients. We present the case of an infant with end-stage kidney disease secondary to ARPKD who developed refractory hypotension following elective bilateral nephrectomies. We describe the use of angiotensin-II infusion with a significant increase in mean arterial blood pressure and successful reduction in other inotropic and vasopressor support. This case suggests that angiotensin-II may represent another valuable therapeutic agent in the treatment of refractory hypotension in anephric infants.

## 1. Introduction

Autosomal recessive polycystic kidney disease is a rare inherited cause of cystic kidney disease leading to renal failure, with mortality rates up to 30% shortly after birth from respiratory insufficiency [1]. The incidence of ARPKD is estimated to be 1:20,000–1:30,000 live births, but it represents an important and common cause of end-stage renal disease that requires renal replacement therapy during early childhood [2]. The clinical course of the disease is highly variable, with most severe cases requiring abdominal decompression early through unilateral or bilateral nephrectomy and peritoneal dialysis (PD) catheter placement for renal replacement therapy [3]. Hypotension is a common complication of nephrectomy, whereas persistent hypotension is a potentially life-threatening complication, particularly after bilateral nephrectomy in young infants [4, 5]. Angiotensin-II is a peptide endocrine hormone with potent vasopressor effect that acts on vascular endothelial receptors. Synthetic human angiotensin-II has been recommended as potential second/third line therapy in the American College of Critical Care Medicine pediatric shock guidelines [6] but its use for refractory hypotension in anephric pediatric patients has only been reported once prior its use in this patient [7].

## 2. Case Presentation

We present an infant of a gravida 1, para 0 mother who was born via planned cesarean section at 34- and 5/7 weeks gestation due to prenatal diagnosis of oligohydramnios at 30 weeks gestation that progressed to anhydramnios at 32 weeks gestation, breech presentation, bilateral enlarged echogenic kidneys concerning for ARPKD, fetal pericardial effusion, and possible coarctation of aorta. Prenatal testing included cell-free DNA screen and viral screening—results of which were unremarkable and screening for autosomal recessive polycystic kidney disease in both parents which yielded father to be a carrier for heterozygous pathogenic variant in PKHD1 gene (c,28811G > A, W937). Mother received full course of antenatal steroids prior to scheduled delivery. A team with complex neonatal resuscitation skills was present at the delivery and the infant was admitted to the neonatal intensive care unit (NICU) for hypoxemic respiratory failure requiring high frequency oscillatory ventilation secondary to pulmonary hypoplasia in the setting of suspected ARPKD.

On the day of birth, a retroperitoneal ultrasound confirmed bilateral microcystic kidney disease with bilateral kidney enlargement (right 9.3 × 5 × 5.9 cm and left 9.5 × 3.1 × 4.6 cm) and urinary bladder was unable to be visualized. A targeted neonatal echocardiogram (TnECHO) was significant for systemic level pulmonary hypertension with normal heart function and outputs, and a persistent ductus arteriosus with bidirectional shunting. Infant was started on inhaled nitric oxide for pulmonary hypertension. Baseline creatinine at 6 h of life was elevated at 1.16 mg/dL.

On postnatal Day 1, the patient underwent peritoneal catheter placement due to anuric state and increasing creatinine (1.59 mg/dL at 16 h). On Day 4, PD was started for worsening generalized edema and systemic hypertension. PD was successful until Day 26 when the PD catheter malfunctioned and would no longer allow for filling or draining of dilysate, presumed to be due to significant nephromegaly and mass effect. A repeat ultrasound at the time was indicative of enlarging kidneys compared to last scan (right 12.4 and left 12.3 cm). In addition, the patient developed bilious emesis and persistent respiratory failure without the ability to wean ventilatory support. It was decided that it was in the patient's best interest, given clinical status, to perform bilateral nephrectomies with placement of hemodialysis catheter (HD) on Day 27 to transition to blood based dialysis. Following the surgical procedure, the infant developed hypotension for which norepinephrine and vasopressin were initiated. Despite these interventions, 24 h after bilateral nephrectomy, the infant had an acute episode of sudden onset hypotension with TnECHO, revealing severe left ventricular dysfunction, treated with epinephrine with subsequent resolution of the dysfunction. The infant continued to have blood pressure lability, which was most predominant while undergoing continuous renal replacement therapy (CRRT). Interventions during this time included fluid resuscitation, frequent adjustment of vasopressors and inotropes, and adjustment on ultrafiltration rate to allow lower net ultrafiltration volume. Despite these interventions, in addition to hydrocortisone and fludrocortisone treating adrenal insufficiency, the infant developed refractory hypotension requiring multiple agents to achieve blood pressure target (> 60–90/30–60 mmHg).

The infants' clinical course was additionally complicated by end-stage renal failure on combination PD and CRRT with ongoing electrolyte imbalances and fluid overload, respiratory failure secondary to pulmonary hypoplasia, staphylococcus epidermidis peritonitis, culture negative sepsis, chylous ascites and pleural effusions with hypoalbuminemia, capillary leak syndrome, and development of clot in HD catheter requiring anticoagulation.

**Figure 1:**
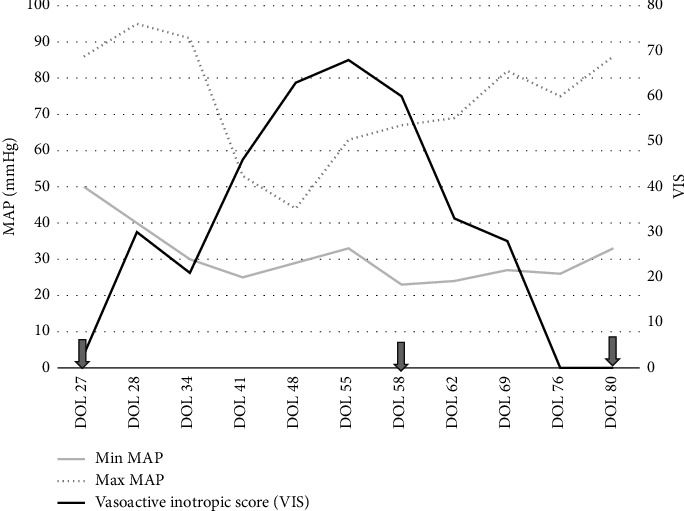
Changes in mean arterial blood pressure (MAP) and vasoactive inotropic score (VIS) in relationship to bilateral nephrectomies and initiation of angiotensin-II infusion. The arrows denote day of bilateral nephrectomies (DOL 27), day of initiation of angiotensin-II infusion (DOL 58), and day of discontinuation of angiotensin-II (DOL 80).

After careful consideration and discussion with the patient's parents, on Day 58, the decision was made to initiate angiotensin-II infusion for management of catecholamine resistant hypotension. On the day of initiation of angiotensin-II infusion, infant's systolic blood pressure was 31–45  mmHg while diastolic blood pressure was 18–27  mmHg and mean blood pressure (MAP) was 23–30  mmHg despite the high dose of vasopressors including dopamine (12 mcg/kg/min), norepinephrine (0.33 mcg/kg/min), vasopressin (1.5 milli-units/kg/min), and intravascular volume resuscitation with intermittent intravenous crystalloid and colloid solutions. The starting dose was chosen based on typical range used in the adult studies as described in ATHOS 3 trial [8]. Infusion concentration was 0.5 mg of angiotensin-II in 50 mL (concentration: 0.01 mg/mL), which correlated to each 0.1 mL/hr of medication being 4 ng/kg/min. The starting rate of angiotensin-II infusion was 20 ng/kg/min, and dose was increased to 24 ng/kg/min after 20 min and to 28 ng/kg/min 30 min after starting infusion. Postinitiation blood pressures were noted to be systolic of 70 s·mmHg over diastolic of 30 mmHg with MAP around 50 mmHg. On the first syringe of use, at a dose of 24 ng/kg/min, there was a 20-point MAP increase, indicating that the use of angiotensin-II was successful in increasing the MAP by at least 10 mmHg, which was the primary endpoint for the use of angiotensin-II in adult studies [8]. Due to the unknown effects of the medication on the pulmonary vascular resistance profile, a TnEcho was obtained within 2 h of initiation and did not show any markers of elevated pulmonary vascular resistance or pulmonary hypertension. Dopamine and vasopressin were able to be discontinued at 15 and 48 h after initiation of angiotensin-II infusion, respectively, due to sustained adequate BP parameters. There was recurrence of hypotension requiring reintroduction of dopamine to stabilize blood pressure on Day 61. The infant remained on angiotensin-II (36–40 ng/kg/min), dopamine (∼8 mcg/kg/min), and norepinephrine (0.3 mcg/kg/min) for one week prior to second attempt at weaning. On Day 68, the infant was weaned off dopamine and on Day 69, weaned off norepinephrine with stable blood pressure and tolerating CRRT. On Day 80, angiotensin-II was weaned off, 22 days after starting its infusion.  Figure 1 portrays the effect of angiotensin-II on vasoactive inotropic score (VIS) before and during infusion. VIS was calculated using the following formula: dopamine dose (μg/kg/min) + dobutamine dose (μg/kg/min) + 100 × epinephrine dose (μg/kg/min) + 100 × norepinephrine dose (μg/kg/min) + 10,000 × vasopressin dose (U/kg/min) + 10 × milrinone dose (μg/kg/min) as defined by Gaies et al. [9]. In summary, dose at initiation of angiotensin-II infusion was 20 ng/kg/min, which was titrated up to maximum dose of 44 ng/kg/min during time on infusion. The angiotensin-II was decreased gradually over 10 days, closely monitoring patient's clinical hemodynamic status. The dose at initiation of wean was 36 ng/kg/min; it was titrated down initially by 4 ng/kg/min every 4–6 h as tolerated. On the last day of wean, dose was 8 ng/kg/min and titrated down by 1 ng/kg/min until 4 ng/kg/min and then turned off.

The patient underwent close clinical monitoring of hemodynamics while receiving angiotensin-II infusion given her clinical history. Despite the initial requirement of nitric oxide on Day 1 for systemic level pulmonary hypertension noted on TnECHO, repeat imaging prior use of angiotensin-II did not reveal concerns for long lasting heart dysfunction, pulmonary disease, or pulmonary hypertension. This remained unchanged while on angiotensin-II with TnECHO on the day of initiation and after stable infusion dose showing stable markers of pulmonary vascular resistance and pulmonary hypertension in addition to no deterioration of heart function parameters or clinical worsening in terms of oxygenation or ventilation parameters. The infant remained on conventional ventilation with minimal changes in ventilator settings and fraction of inspired oxygen 0.3–0.4.

## 3. Discussion

We hereby present the first case in which angiotensin-II was successfully used for persistent refractory hypotension postbilateral nephrectomies in an infant with prenatal diagnosis of ARPKD. The patient presented with refractory hypotension despite adequate vasopressor and inotrope support, multiple adjustments and attempts to improve intravascular volume, and modifications to the CRRT regimen.

Blood pressure regulation is maintained by neural mechanisms of the autonomic nervous system and humoral mechanisms of the kidneys and pituitary gland consisting, mainly of the renin–angiotensin aldosterone system (RAAS) and vasopressin [10]. The RAAS is a crucial mediator of cardiac, vascular, and renal physiology through the regulation of vascular tone, salt, and water homeostasis [11]. Renin is initially synthesized by renal and nonrenal tissues as prorenin, which is then converted to active renin solely in the juxtaglomerular cells of the kidney [12]. Angiotensin-II is the primary mediator of the physiologic effects of RAAS, including blood pressure, volume regulation, and aldosterone secretion [13]. The physiological effects of endogenous angiotensin-II on extracellular volume and blood pressure are mediated by peripheral vasoconstriction of arteriolar vascular smooth muscle, aldosterone secretion, and sodium reabsorption, increasing sympathetic outflow from central nervous system and release of vasopressin from hypothalamus [11]. Previous research shows that age and plasma renin levels have an inverse relationship with neonates having higher concentrations than older children. In an investigative review in 2019, Suessenbach et al. reported that age has a major impact on circulating AngI, AngII, and Ang1-7, especially in the newborn period with decreasing values from newborn to adolescents and although their findings indicated high variability, the overall trend was for lower concentrations with increasing age [14]. There have been no studies evaluating the trajectory of extrarenal prorenin production and it is not currently well understood if there is a point in which extrarenal sources are sufficient for hemodynamic stability. Thus, it was hypothesized that as the juxtaglomerular cells in the kidney are the primary source of conversion of prorenin to renin, leading to activation of the RAAS pathway, after nephrectomy, there would be insufficient activation of the RAAS pathway and thus angiotensin-II infusion would be an adequate treatment for this patient.

In December 2017, the U.S. Food and Drug Administration (FDA) approved the use of Giapreza, a synthetic form of human angiotensin-II, as a vasoconstrictor for intravenous infusion to increase blood pressure in adults with septic or other distributive shock. This was in response to the ATHOS-3 trial, a multinational, randomized, double blind study in which adults with septic or other distributive shock who remained hypotensive despite fluid and vasopressor therapy. These patients had a significant increase in mean arterial pressure after receiving Giapreza compared with those who received placebo [8].

Although not yet approved for use in children, to date, previous case reports have described the benefits of the use of synthetic angiotensin-II in the pediatric population. Bailey et al. described two patients with septic shock requiring high-dose vasopressors who were treated with angiotensin-II as part of an open label study. Both patients had a significant increase in mean arterial pressure within 90 and 120 min of initiation of angiotensin-II, with a reduction of the dose of catecholamines and vasopressin infusions [7]. Razdan et al. described the use of angiotensin-II in a critically ill infant with refractory hypotension secondary to congenital renal agenesis [12]. In 2023, Tezel et al. described a case series of utilization of angiotensin-II in 23 critically ill patients ranging from 1.5 months to 23 years, none with congenital kidney disease, with catecholamine-resistant vasodilatory shock and reported elevated renin values pre angiotensin-II initiation in 11 (47%) of them [15].

Our case is the first in the literature to describe the use of synthetic angiotensin-II to manage refractory hypotension in a patient with ARKPD status postbilateral nephrectomies in the neonatal period. We present this case to add to the literature illustrating the potential benefit of its use in infants or pediatric patients with refractory hypotension. Bilateral nephrectomy may lead to an abrupt disruption of the RAAS, which is crucial for blood pressure regulation and there is paucity of knowledge of the RAAS pathway in infants with congenital kidney disease or those who undergo bilateral nephrectomies. We suspect young infants are at higher risk for developing persistent hypotension due to a deficiency of renin after bilateral nephrectomy, thought to be due to infant's normally having a highly active RAAS [4] and to the major source of renin early in life being the kidneys.

Other aspects to be considered prior to initiation of angiotensin-II infusion is the need for central venous access, as it has the potential to cause severe vasoconstriction of the peripheral vessels and the concurrent deep vein thrombosis prophylaxis due to increased risk of both arterial and venous thromboembolic events [16].

## 4. Conclusions

Persistent refractory hypotension is a potentially life-threatening complication of nephrectomies in the pediatric population. The use of synthetic angiotensin-II infusion had a significant improvement in mean arterial pressure in this patient with no impact on pulmonary vascular resistance or development of pulmonary vascular disease in a critically ill infant with refractory hypotension status postbilateral nephrectomy secondary to ARPKD, allowing for weaning off other vasopressors and inotropes. This case report highlights the need for further studies to better understand the RAAS pathway in infants with congenital kidney disease or those who undergo bilateral nephrectomies.

### 4.1. Future Directions

Further research regarding the indications, dosing, efficacy of treatment, and plan for discontinuation would be beneficial for understanding the mechanism of action and benefits of the use of angiotensin-II infusion in this specific population of patients. Advanced hemodynamics monitoring and surveillance of pulmonary hemodynamics with targeted neonatal echocardiogram must be considered while undergoing angiotensin-II infusion given the drug's potential impact on pulmonary vasculature, pulmonary vascular resistance, and pulmonary hypertension.

## Data Availability

The data that support the findings of this study are available on request from the corresponding author.
